# COVID-19 Chest Computed Tomography to Stratify Severity and Disease Extension by Artificial Neural Network Computer-Aided Diagnosis

**DOI:** 10.3389/fmed.2020.577609

**Published:** 2020-12-04

**Authors:** Alysson Roncally S. Carvalho, Alan Guimarães, Gabriel Madeira Werberich, Stephane Nery de Castro, Joana Sofia F. Pinto, Willian Rebouças Schmitt, Manuela França, Fernando Augusto Bozza, Bruno Leonardo da Silva Guimarães, Walter Araujo Zin, Rosana Souza Rodrigues

**Affiliations:** ^1^UnIC, Faculty of Medicine, Cardiovascular R&D Center, Centro Hospitalar Universitário Do Porto (CHUP), Porto University, Porto, Portugal; ^2^Laboratory of Pulmonary Engineering, Biomedical Engineering Program, Alberto Luiz Coimbra Institute of Post-Graduation and Research in Engineering, Universidade Federal do Rio de Janeiro, Rio de Janeiro, Brazil; ^3^Laboratory of Respiration Physiology, Carlos Chagas Filho Institute of Biophysics, Universidade Federal do Rio de Janeiro, Rio de Janeiro, Brazil; ^4^Department of Radiology, Universidade Federal do Rio de Janeiro, Rio de Janeiro, Brazil; ^5^Hospital Barra D'Or, Rio de Janeiro, Brazil; ^6^National Institute of Infectious Disease, Oswaldo Cruz Foundation (INI/Fiocruz), Rio de Janeiro, Brazil; ^7^Radiology Department, Centro Hospitalar Complexo Universitário Do Porto (CHUP), Porto, Portugal; ^8^Instituto de Ciências Biomédicas Abel Salazar (ICBAS), Porto University, Porto, Portugal; ^9^IDOR - D'Or Institute for Research and Education, Rio de Janeiro, Brazil; ^10^Hospital Niterói D'Or, Rio de Janeiro, Brazil

**Keywords:** COVID-19 pneumonia, radiomics, computer-aided diagnosis, deep learning, quantitative chest CT-analysis

## Abstract

**Purpose:** This work aims to develop a computer-aided diagnosis (CAD) to quantify the extent of pulmonary involvement (PI) in COVID-19 as well as the radiological patterns referred to as lung opacities in chest computer tomography (CT).

**Methods:** One hundred thirty subjects with COVID-19 pneumonia who underwent chest CT at hospital admission were retrospectively studied (141 sets of CT scan images). Eighty-eight healthy individuals without radiological evidence of acute lung disease served as controls. Two radiologists selected up to four regions of interest (ROI) per patient (totaling 1,475 ROIs) visually regarded as well-aerated regions (472), ground-glass opacity (GGO, 413), crazy paving and linear opacities (CP/LO, 340), and consolidation (250). After balancing with 250 ROIs for each class, the density quantiles (2.5, 25, 50, 75, and 97.5%) of 1,000 ROIs were used to train (700), validate (150), and test (150 ROIs) an artificial neural network (ANN) classifier (60 neurons in a single-hidden-layer architecture). Pulmonary involvement was defined as the sum of GGO, CP/LO, and consolidation volumes divided by total lung volume (TLV), and the cutoff of normality between controls and COVID-19 patients was determined with a receiver operator characteristic (ROC) curve. The severity of pulmonary involvement in COVID-19 patients was also assessed by calculating *Z* scores relative to the average volume of parenchymal opacities in controls. Thus, COVID-19 cases were classified as mild (<cutoff of normality), moderate (cutoff of normality ≤ pulmonary involvement < *Z* score 3), and severe pulmonary involvement (*Z* score ≥3).

**Results:** Cohen's kappa agreement between CAD and radiologist classification was 81% (79–84%, 95% CI). The ROC curve of PI by the ANN presented a threshold of 21.5%, sensitivity of 0.80, specificity of 0.86, AUC of 0.90, accuracy of 0.82, *F* score of 0.85, and 0.65 Matthews' correlation coefficient. Accordingly, 77 patients were classified as having severe pulmonary involvement reaching 55 ± 13% of the TLV (*Z* score related to controls ≥3) and presented significantly higher lung weight, serum C-reactive protein concentration, proportion of hospitalization in intensive care units, instances of mechanical ventilation, and case fatality.

**Conclusion:** The proposed CAD aided in detecting and quantifying the extent of pulmonary involvement, helping to phenotype patients with COVID-19 pneumonia.

## Introduction

Chest computed tomography (CT) has been widely used to assess COVID-19 pneumonia and is a key tool for the detection of lung abnormalities and for evaluating the extension and severity of pulmonary involvement (PI) (1, 2).

Patients with COVID-19 usually exhibit radiological patterns classified as ground-glass opacity (GGO), crazy paving (CP), linear opacities (LO), and consolidation (1, 2). Reticular opacities (RO), characterized by coarse linear, curvilinear opacities, fibrotic streaks, and subpleural lines, may be seen in late phases, often associated with GGO and parenchymal distortion (1, 3, 4).

Some attempts have been made to achieve an automatic quantification of PI in chest CT, most of them based on texture analysis techniques. Classic statistical methods may include the parameters of first-, second-, and third-order statistics and other composite or custom-made texture parameters (5–7). Their disadvantages include the extensive training required before automated or semiautomated segmentation, evaluation of the potential usefulness of a particular parameter only after implementation, and cumbersome and time-consuming adaptation to new segmentation tasks.

In the present study, we propose a method for objective and automated quantification and classification of COVID-19-related pneumonia from chest CT using a simple computer-aided diagnosis (CAD) system, considering the different radiological CT lung patterns commonly used in clinical practice. Determining the quantity, type, and distribution of abnormalities by an automated tool should prove helpful in clinical practice by aiding in noninvasive diagnostic determination, detecting change in disease over symptom onset, and stratifying the risk of hospitalization in the intensive care unit (ICU), the necessity of mechanical ventilation, or case fatality.

## Materials and Methods

### Study Design and Patients

**Figure 1:**
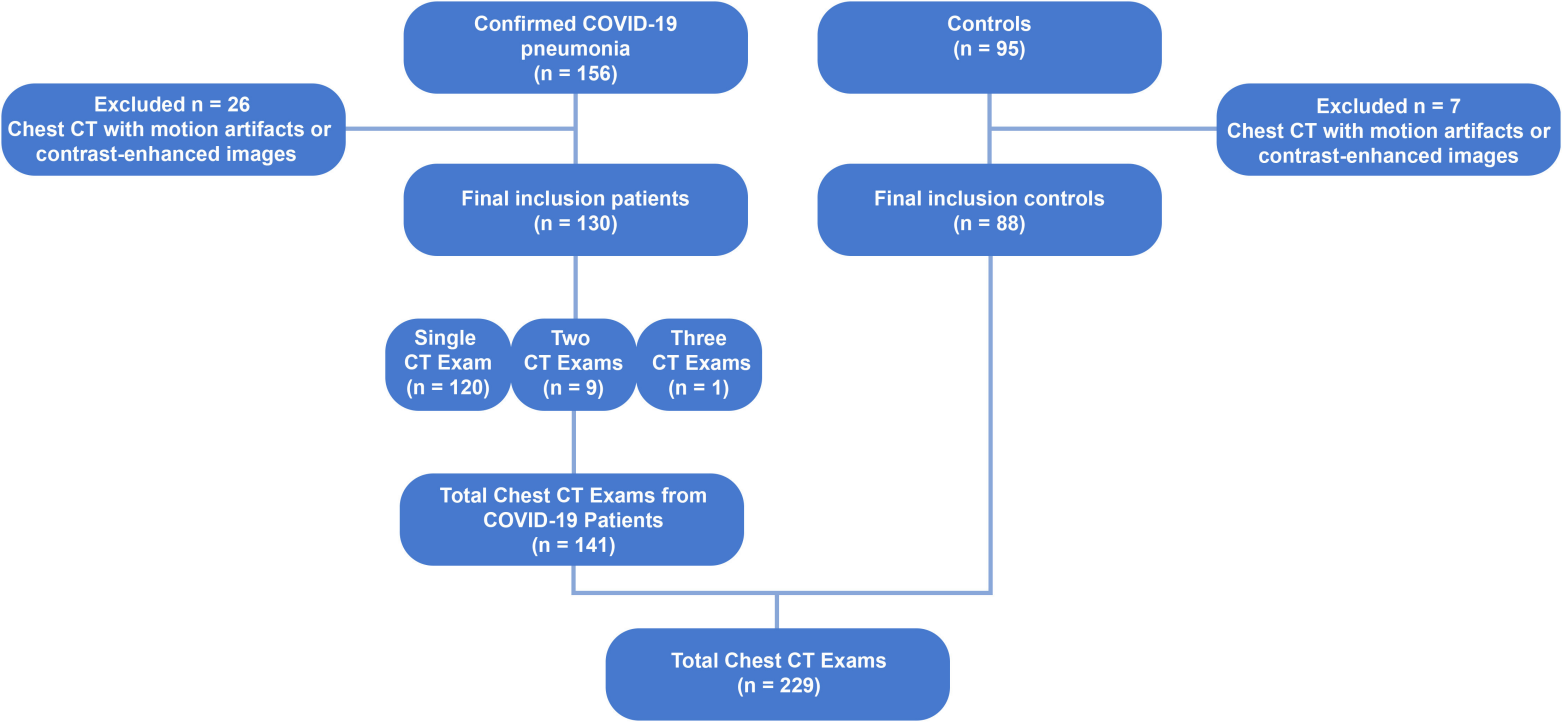
Flow chart diagram showing the patient enrollment and computed tomography selection process. CT, computed tomography; COVID-19, corona virus disease-19; RT-PCR, reverse-transcription polymerase chain reaction.

One hundred thirty consecutive patients with COVID-19, confirmed with reverse-transcription polymerase chain reaction (RT-PCR) for COVID-19 in nasal-pharyngeal swab, admitted to three hospitals between April to June 2020, who underwent chest CT scans and presented with pneumonia, were retrospectively studied. Ten patients underwent more than one CT scan, totaling to 141 scans. The chest CTs of 88 healthy subjects served as controls. Scans with severe motion artifacts and contrast-enhanced scans were excluded. Figure 1 shows the patient enrollment and CT scan selection flow chart.

The Research Ethics Committee approved the study that complied with the current national and international standards.

### Clinical and Laboratory Data, Definitions, and Outcomes

The clinical and laboratory findings of each patient were recorded at admission. CT was performed within 12 h after the clinical evaluation and laboratory findings.

Serum C-reactive protein concentration collected at the admission was used as a marker of systemic inflammation. ICU admission, invasive ventilation, and in-hospital case fatality were considered as our clinical outcomes.

### Chest Computed Tomography Acquisition

CT scans were performed on a 64-channel multislice (Brilliance 40 scanner, Philips Medical Systems, Cleveland, OH, USA, and General Electrics Lightspeed VCT, Chicago, IL, USA), a 128-channel multislice dual-source CT system (Somatom Definition Flash, Siemens, Forchheim, Germany), or a 16-channel multislice (Emotion 16 CT, Siemens, Erlangen, Germany). The acquisitions were gathered with the patients in supine position with 120 kV and 120–300 mA, slice thickness ranging from 1 to 2 mm with 50% superposition, and 512 × 512, 768 × 768, or 1,024 × 1,024 voxels matrix.

Reconstruction algorithms were B50f (49 subjects), B60f (1), B70s (17), C (1), FC13 (5), FC86 (2), I50f2 (1), L (80), LUNG (69), and SOFT (4), depending on the CT manufacture.

### Lung and Airway Segmentation

The lung parenchyma and the airways were segmented from chest CT scans using the Region Growing algorithm using the module Chest Imaging Platform (3D Slicer version 4.8.1). Then, the region of interest (ROI) selected by the algorithm was edited by visual inspection (8). Thereafter, all images were exported to an in-house-developed software (Quantitative Lung Image, QUALI) written in MATLAB® (MathWorks®, Natick, MA, USA), and airways were subtracted from the lung ROI.

### Image Rescaling

After segmentation, CT images were rescaled so that images from different CT scans could be comparable. For that purpose, a circular ROI was positioned outside the body of the individuals (HU_Air_) and another, with the same shape and area, was positioned in the descending aorta (HU_Aorta_).

Then, the average density value present within both ROIs was calculated, and a linear regression was performed, using the least squares method, using the mean HU_Air_ and HU_Aorta_ values as dependent variables and −1,000 and +50 HU, respectively, as independent variables. Scaling was performed by multiplying all voxels present in the lung parenchyma by the angular coefficient together with the addition of the intercept (9, 10). Both the selection of the ROIs and the scaling of the voxels were carried out using QUALI Software.

### Visual Classification of Radiological Patterns in COVID-19 CT Scans

Two chest radiologists blinded to patient identification, clinical data, and outcomes independently selected up to four ROI per COVID-19 patient visually classified as well-aerated regions, GGO, CP/LO, and consolidation. The ROI consisted of a circle with a fixed radius of 4 mm, with a spanning area of about 30 voxels in each CT section.

### Development of the Supervised Neural Network Architecture

From all ROIs belonging to the same radiological pattern class, a density histogram was calculated, and the respective quantiles (2.5, 25, 50, 75, and 97.5%) were used to train a supervised artificial neural network (ANN). Initially, the number of ROIs consensually assigned to well-aerated regions (472 ROIs), GGO (413 ROIs), CP/LO (340 ROIs), and consolidation (250 ROIs), totaling 1,475 ROIs, were balanced by the lowest number of ROIs (250 ROIs). Thus, 1,000 ROIs were used for ANN training (700 ROIs), validation (150 ROIs), and test (150 ROIs). In order to keep the same ROIs for ANN architecture assessment, ROIs were drawn to undergo training, validation, and test only once since the same groups were used in each training, validation, and test session. No feature scale was necessary since all data are expressed in the same scale in Hounsfield units and the training algorithm used the scaled conjugate gradient backpropagation (11). The training stopped when the validation error increased for six iterations, and the best validation performance was obtained based on the minimization of the cross-entropy.

Several architectures were tested with a single hidden layer with 20 up to 100 neurons. The overall and intraclass agreement of the balanced test confusion matrix between each respective ROI quantile consensually classified by radiologists and by the ANN classifier was assessed and used to define the best ANN architecture.

To evaluate the ANN classifier's final performance, the confusion matrix, the receiver operator characteristic (ROC) curve for each class, and the relationship between cross-entropy and ANN epochs in training, validation, and test sets were assessed.

**Figure 2:**
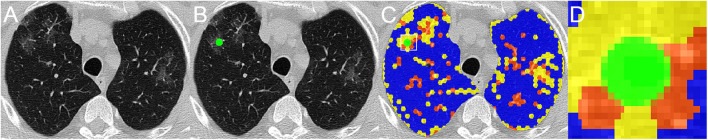
A representative CT scan axial image of a COVID-19 patient **(A)**, the region of interest (ROI), in green, classified by one radiologist as ground glass opacities (GGO) **(B)**, and the overlapping of radiologist's ROI and the grid of regular hexagons with radii of 4 mm **(C)**, with each grid already classified as well-aerated regions (blue), GGO (yellow), and CP/LO (orange) areas. **(D)** Amplification of the overlapped region marked with the white square in **(C)**. Note that, within ROI, there are voxels of two different classes, GGO in yellow and CP/LO in orange. For comparison purposes, the ROI region is classified as the most prevalent voxel class, in this case as GGO class.

Having established the qualitative equivalence of ANN classifier and expert groupings, all results were verified with expert visual validation (Figure 2). Additionally, a grid of regular hexagons with radii of 2, 3, and 4 mm, accounting for 12 up to 42 voxels, was created. In the overlapping region of each radiologist ROI and hexagons (Figure 2C), the local histogram quantiles were computed from the voxels contained in the hexagon and served as an impute to ANN classifier. The best hexagon dimension was computed with the unweighted Cohen's kappa test between the ANN classification and their respective ROI classification attributed by the radiologist. In the assessment of the overlapped regions by radiologist's ROI, if there were hexagons belonging to different classes into a given ROI, the most prevalent classification would be used as the final classification of the voxels for comparison purposes (Figure 2D).

### CT Scan Quantitative Analysis and CAD Report

The previously defined regular hexagon grid was used to group voxels in the whole-lung CT scan. The number of voxels belonging to each of the parenchymal classes was calculated across the whole lungs. The voxels identified as vessels were included as normal to account for the total lung volume.

Total lung volume (TLV), i.e., the sum of air plus tissue volume, was calculated as:

(1) $$\begin{array}{l} \text{TLV}\left(\text{ml}\right)={\left(\text{pixel size}\right)}^{2}\;\times \text{ slice thickness}\\ \;\times \text{ total number of pixels of the whole lung} \end{array}$$

Lung weight, in grams, was calculated as:

(2) $$\begin{array}{l} \text{Lung weight }\left(\text{g}\right)=\left[\left(\text{HU}-\text{H}{\text{U}}_{\text{Air}}\right)/\left(\text{H}{\text{U}}_{\text{Aorta}}-\text{H}{\text{U}}_{\text{Air}}\right)\right]\\ \;\times \text{voxel volume}\times 1.04\text{ g}/\text{ml} \end{array}$$

where 1.04 mg/ml means lung tissue density, and HU is voxel density in HU scale (9).

### Determination of PI

After CAD classification of parenchymal opacities, the extent of PI was calculated as the cumulative volumetric sum of GGO, CP/LO, and consolidation adjusted to TLV.

The threshold of parenchymal opacities between controls and COVID-19 was determined with a ROC curve from the histogram of parenchymal opacities in the control group and COVID-19 patients. The area under the ROC curve (AUC) was calculated, and the threshold sensibility, specificity, accuracy, positive and negative predictive values, *F* score (a measure of ANN's precision and recall balance), and Matthews correlation coefficient were also computed.

To evaluate the severity of PI in COVID-19, we used the *Z* score in relation to the average volume of lung parenchyma opacities in the control group. Thus, the *Z* score was used to describe the position of the calculated volume of pneumonia in COVID-19 patients in terms of its distance from the mean calculated volume of GGO plus CP/LO and consolidation in the control group. This distance is expressed in terms of standard deviation units. Accordingly, the *Z* score of COVID-19 patients is positive if the value lies above the mean volume of GGO, CP/LO, and consolidation in the control group and negative if it lies below it.

Thereafter, patients with COVID-19 were classified as having mild (PI < ROC threshold), moderate (ROC threshold ≤ PI < 3 *Z* score), or severe (PI ≥ 3 *Z* score) PI.

### Statistical Analysis

The normality of the data (Kolmogorov–Smirnov test with Lilliefors' correction) and the homogeneity of variances (Levene median test) were tested. Since both conditions were always satisfied, all data are presented as mean and standard deviation.

A one-way ANOVA test followed by Bonferroni's *post hoc* test assessed the statistical differences among patients with mild, moderate, and severe COVID-19 pneumonia. A *p*-value < 0.05 was defined as statistically significant. All statistical analyses were performed using Matlab® software (MathWorks®, Natick, MA, USA).

## Results

**Figure 3:**
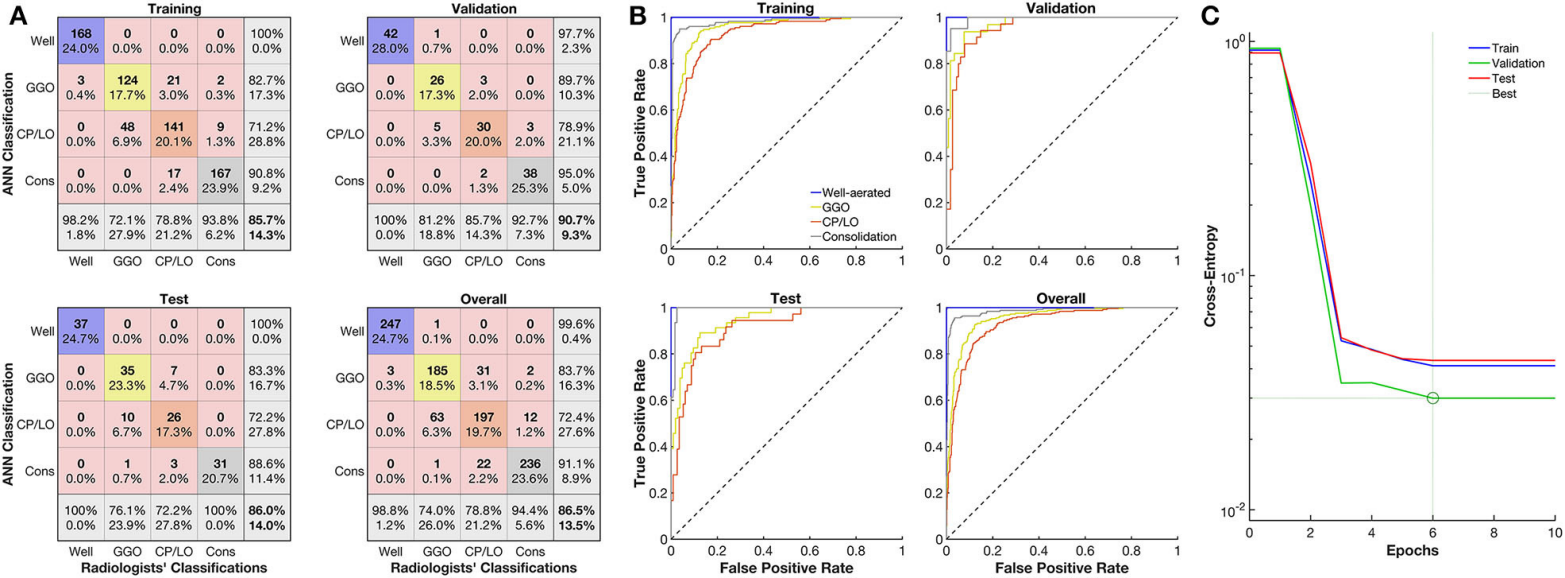
Evaluation of the artificial neural network (ANN) classifier performance. **(A)** Confusion matrix from the comparison between the ANN and radiologists' classification of well-aerated (blue line), ground glass opacities (GGO, yellow line), CP/LO (orange), and consolidation (gray) in training (first row and column), validation (first row, second column), test (second row, first column), and overall (second row, second column) sets. **(B)** Each respective receiver operator characteristic curve for well-aerated regions (blue line), GGO (yellow line), CP/LO (orange) and consolidation (gray) ANN classification in training (first row and column), validation (first row, second column), test (second row, first column), and overall (second row, second column) sets. **(C)** Cross-entropy at each epoch in training (blue line), validation (green line), and test (red line) sets. Dotted lines represent the best validation performance determined based on the minimization of the cross-entropy at epoch 6.

The ANN architecture with a single hidden layer of 60 neurons showed the best agreement in the confusion test matrix among the other architectures tested, with an overall agreement of 86% being 100% for well-aerated regions, 76% for GGO, 72% for CP/LO, and 100% for consolidation (Figure 3A). The architecture with 40 and 100 neurons presented overall agreements of 66 and 74%, respectively. Despite that the architecture with 20 and 80 neurons presented a similar overall agreement, a reduction in the agreement of well-aerated regions (from 100 to 98 and 95%) and consolidation (from 100 to 95 and 94%) was observed in 60-, 80-, and 20-neuron architectures, respectively. No improvement in the performance of the ANN classifier was observed with the addition of a second neuron layer. The ROC curve from each radiological pattern is presented in Figure 3B. The classifier performance was much better for well-aerated regions and consolidation, with an AUC of 1.00 and 0.99, respectively. The performance for GGO and CP/LO, despite being lower, was quite acceptable with an AUC of 0.94 and 0.91, respectively. The best validation performance occurred at epoch 6 (Figure 3C).

**Figure 4:**
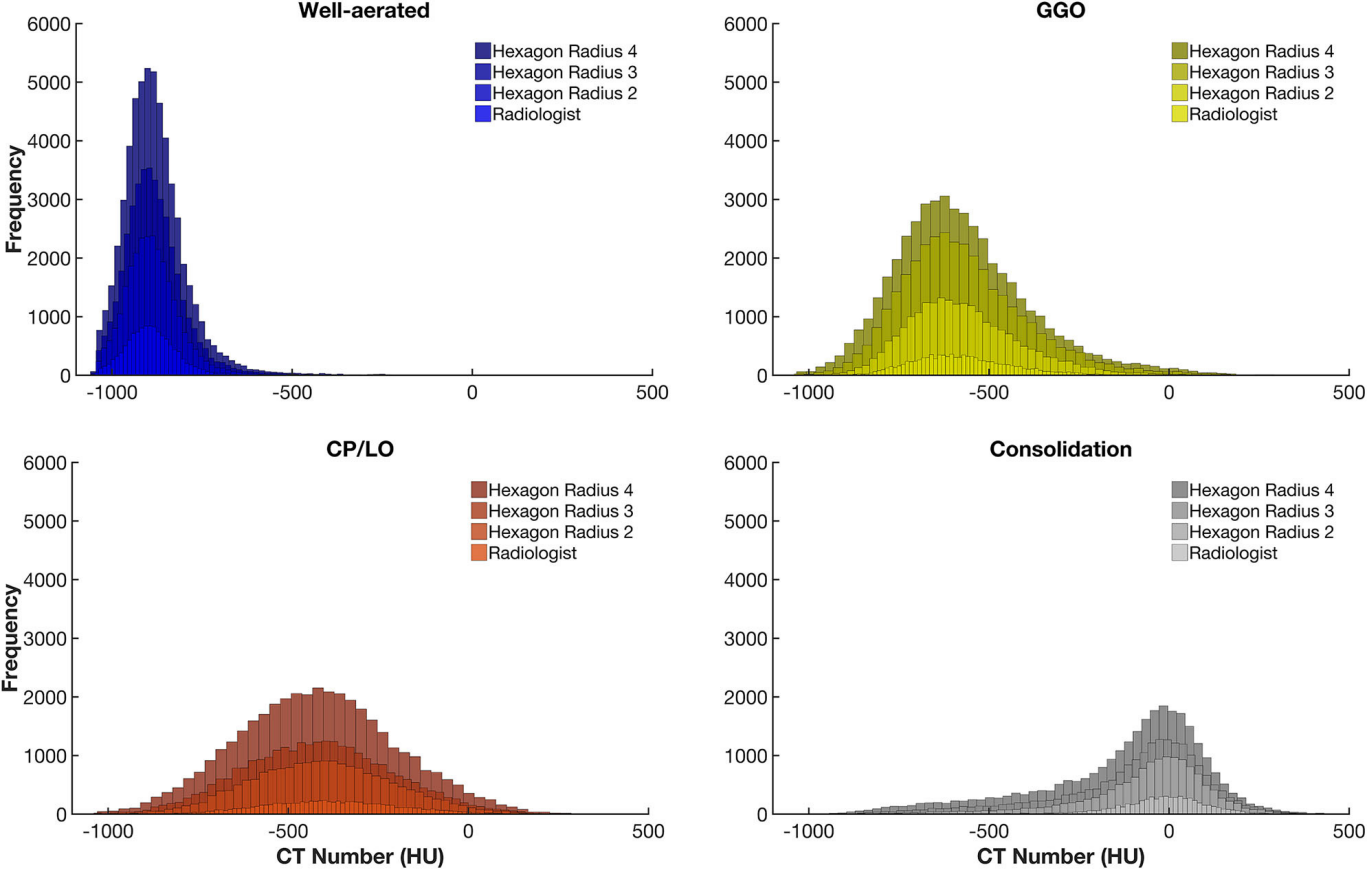
Frequency histograms of the densities expressed in Hounsfield units from all regions of interest visually assigned as well-aerated regions (colored in blue), ground glass opacities (GGO, yellow), crazy paving and linear opacities (CP/LO, orange), and consolidation (light gray). Note that, with the increase in the radius of the regular hexagon, there is an increase in the number of voxels and that, although there is no significant change in the average density values, there is an important increase in the dispersion of voxel densities. This appears to contribute to a reduction in the artificial neural network classifier performance.

The CT density histogram of ROIs visually assigned as well-aerated regions, GGO, CP/LO, and consolidation in COVID-19 patients is depicted in Figure 4. Additionally, the histograms from overlapped regions with regular hexagons with radii of 2, 3, and 4 mm are also presented. The best overall agreement between the neural network and the radiologists' ROIs occurred with the 2-mm-radius hexagon with an unweighted Cohen's kappa of 81% (79–84%, 95% CI) when compared to the hexagons of 3-mm radius (76%, 79–84%, 95% CI) and 4-mm radius (72%, 79–84%, 95% CI). Thus, the 2-mm-radius hexagon presented an agreement of 98% for well-aerated regions, 81% for GGO, 70% for CP/LO, and 93% for consolidation (Table 1).

**Table 1:** Agreement between all (1,475) consensual radiologists' regions of interest (columns) and the supervised neural network (ANN) classifier with the regular hexagon grid of radii 2 (rows) for the 1,000 ROIs used to train, test, and validate Quantitative Lung Image Deep Learning software.

		**Consensual radiologists' classification**			
		**Well aerated**	**GGO**	**CP/LO**	**Consolidation**
	Well aerated	**464 (98%)**	1	0	0
ANN classifier	GGO	7	**335 (81%)**	68	2
	CP/LO	1	76	**239 (70%)**	16
	Consolidation	0	1	33	**232 (93%)**

*The overall strength of agreement as evaluated by Cohen's kappa test was 81% (79–84%, 95% CI). Data between parentheses are the agreement for each radiological pattern. Bold values represent concordance between consensus radiology and ANN classifier*.

**Figure 5:**
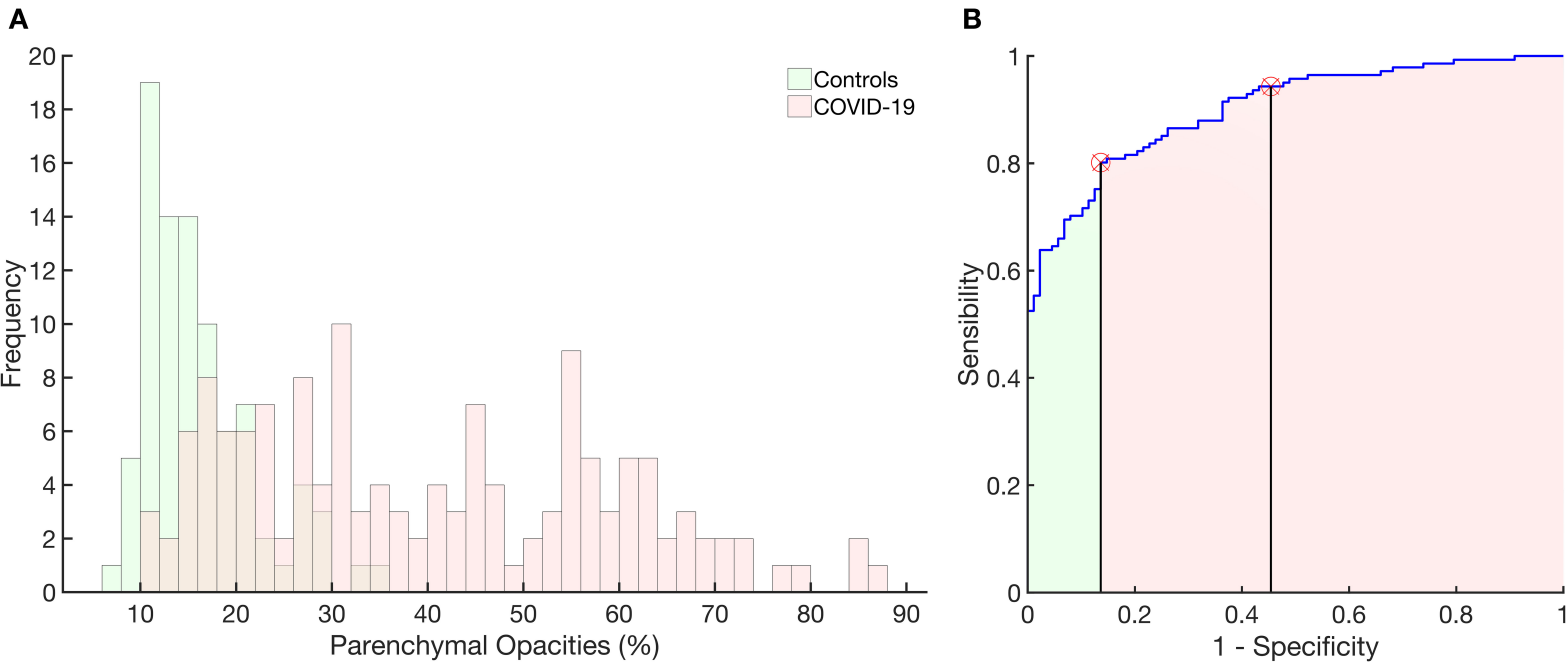
Histograms of the frequency of occurrence of parenchymal opacities in the control group (light green) and in patients with COVID-19 (pink). Right panel: receiver operator characteristic curve with the area under the curve (AUC) hatched in light green and pink. The vertical lines mark the normality cutoff (equivalent to 21% of total lung volume represented by pulmonary opacities) and the Z score = 3 (equivalent to 33% of the total lung volume and used to classify SSc patients as with severe pulmonary involvement). Between the normality cutoff up to Z score < 3, COVID-19 patients were classified as with moderate pulmonary involvement. The use of parenchymal opacities as an indicator of pulmonary involvement presented 0.80 sensitivity, 0.86 specificity, with an AUC of 0.90, accuracy of 0.82, 0.90 positive predictive value and 0.73 negative predictive value, *F* score of 0.85, and Matthews correlation coefficient of 0.65.

Figure 5A presents the histogram of parenchymal opacities in controls and COVID-19 patients. Accordingly, the ROC curve determined an optimal threshold of 21.5%, with sensitivity of 0.80, specificity of 0.86, AUC of 0.90, accuracy of 0.82, *F* score of 0.85, and Matthews' correlation coefficient of 0.65 (Figure 5B).

**Figure 6:**
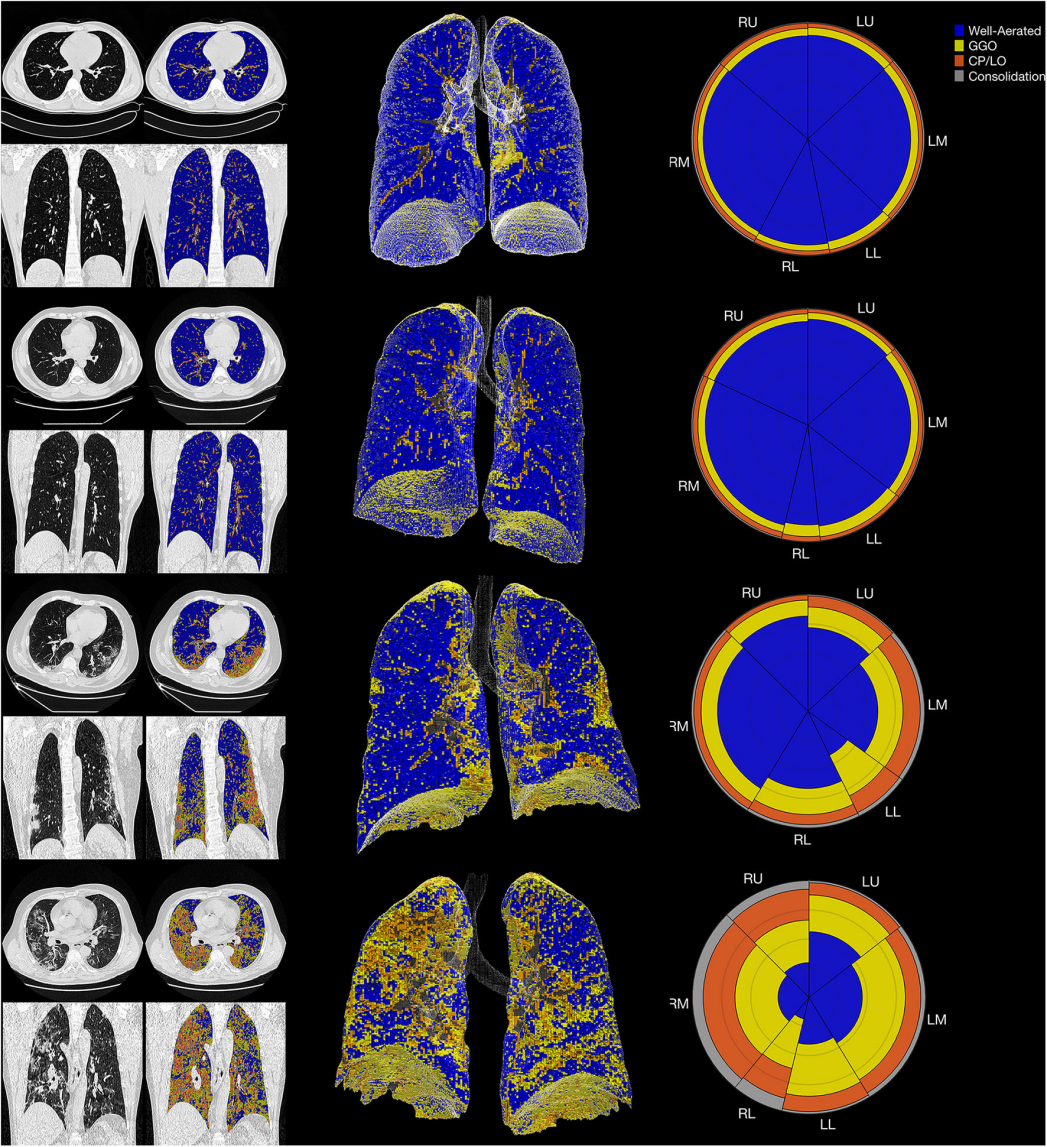
The summary glyph (right column) to the underlying 3D scan data (middle column) and CT images in axial and coronal slices (left column) in a representative case from the control group (row A) and COVID-19 with mild (row B), moderate (row C), and severe (row D) pneumonia involvement. In the glyph (right column), the first letter (R/L) indicates the right and left lung, the second letter (U/M/L) denotes, respectively, the upper, middle, and lower lung zones.

In controls, the volume related to all parenchymal opacities was 16 ± 6%, with being 12 ± 6% classified as GGO, 3 ± 1% as CP/LO, and 1 ± 0.3% as consolidation that represent small bronchi and peribronchial vessels and possible partial volume effects of pleural or diaphragm interfaces as can be seen in Figure 6 (uppermost panels).

The final CAD reports from four representative subjects (control, mild, moderate, and severe COVID-19 PI) are presented in Figure 6.

Seventy-seven chest CT images of COVID-19 patients were classified as presenting severe PI (55 ± 13% of the TLV with GGO + CP/LO + consolidation), while 36 (25%) and 28 (20%) were classified as just moderate (27 ± 4%) or mild (17 ± 3%) (Table 2). In patients with severe PI, pneumonia was mainly characterized by GGO (35 ± 10%) and CP/LO (14 ± 7%) with just 5 ± 4% of the TLV being assigned as consolidation. Furthermore, the degree of PI was fairly different from that observed in patients with moderate or mild PI (*p* < 0.001) (Table 2).

**Table 2:** COVID-19 and controls demographic and computer-aided diagnosis (CAD) quantitative data, laboratory parameters and clinical outcomes.

	**COVID-19 *N* = 130**			**Controls *N* = 88**	***P* value**
	**Severe *N* = 71**	**Moderate *N* = 32**	**Mild *N* = 27**		
**Demographic data**					
Sex (male/female)	51/20	20/12	16/11	24/64	
Age (years)	65 ± 16	58 ± 15	54 ± 17	59 ± 21	0.006^b^
BMI (kg/m^2^)	28 ± 5	29 ± 5	27 ± 5	26 ± 4	
CAD data^*^	N = 77	N = 36	N = 28		
Total lung volume (ml)	3,639 ± 931	4,905 ± 782	5,266 ± 1,030	4,411 ± 1,035	<0.001^a,b^
Lung weight (g)	1,037 ± 251	928 ± 151	795 ± 152	628 ± 166	<0.001^b^ 0.03^a,c^
Pulmonary involvement (%)	55 ± 13	27 ± 4	17 ± 3	16 ± 6	<0.001^a,b,c^
Well aerated (%)	45 ± 13	73 ± 4	83 ± 3	84 ± 6	<0.001^a,b,c^
GGO (%)	35 ± 10	20 ± 3	11 ± 3	12 ± 6	<0.001^a,b,c^
CP/LO (%)	14 ± 7	6 ± 2	4 ± 1	3 ± 1	<0.001^a,b^
Consolidation (%)	5.0 ± 4	1.5 ± 0.5	1.0 ± 0.2	1.0 ± 0.3	<0.001^a,b^
**Laboratory data**	N = 71	N = 32	N = 27		
White blood count (×10^3^/μl)	6.4 ± 3.0	4.4 ± 1.9	5.6 ± 1.5	–	0.002^a^
Lymphocytes count (×10^3^/μl)	1.2 ± 1.1	1.0 ± 0.7	1.3 ± 0.6	–	
Lactate dehydrogenase (U/L)	334 ± 161	260 ± 159	266 ± 112	–	
CRP (mg/L)	64 ± 80	23 ± 74	4 ± 5	–	0.03^a^ <0.001^b^
GOT (U/L)	44 ± 30	34 ± 22	33 ± 21	–	
GPT (U/L)	41 ± 33	37 ± 33	31 ± 28	–	
Creatinine (mg/dl)	1.0 ± 0.4	1.2 ± 1.6	1.0 ± 0.3	–	
**Clinical outcome**	N = 71	N = 32	N = 27		
Symptom onset (days)	7.7 ± 4.6	5.1 ± 3.4	6.0 ± 4.4		0.015^a^
ICU (%)	27%	19%	22%	–	
i-MV (%)	14%	9%	15%	–	
Case fatality (%)	11%	6%	0%		

a *Severe vs. moderate*.

b *Severe vs. mild*.

c *Moderate vs. mild*.

*Data are shown as mean ± one standard deviation. Significant p-values (<0.05) are shown in italics*.

*CRP, C-reactive protein; GOT, glutamic oxaloacetic transaminase; GPT, glutamic pyruvic transaminase; TLV, total lung volume (in ml); lung weight, whole lung tissue fraction (in g); GGO, volume related to ground glass opacities adjusted to TLV; CP/LO, volume related to crazy paving and linear opacities adjusted to TLV; consolidation, volume related to consolidation adjusted to TLV; CRP, serum C-reactive protein concentration; ICU, intensive care unit; i-MV, invasive mechanical ventilation*.

* *Data from COVID-19 refer to 141 CT scan series*.

COVID-19 patients classified as having a severe PI were older than those with mild PI (65 ± 16 vs. 54 ± 17 years, respectively) and presented a significant reduction in TLV (3,639 ± 931 vs. 4,890 ± 704 in moderate and 5,247 ± 1,067 ml in mild COVID-19 pneumonia), significant increase in lung weight (1,037 ± 251 vs. 935 ± 143 and 799 ± 159 g, respectively), and higher serum C-reactive protein concentrations at admission (62 ± 79 in severe vs. 23 ± 73 in moderate and 4 ± 5 mg/dl in mild COVID-19 pneumonia). Moreover, a higher prevalence of hospitalization in ICU (31 vs. 20 and 21%), necessity of mechanical ventilation (18 vs. 9 and 14%), and case fatality (11 vs. 6 and 0%) was observed when compared to moderate and mild PI, respectively (Table 2).

**Figure 7:**
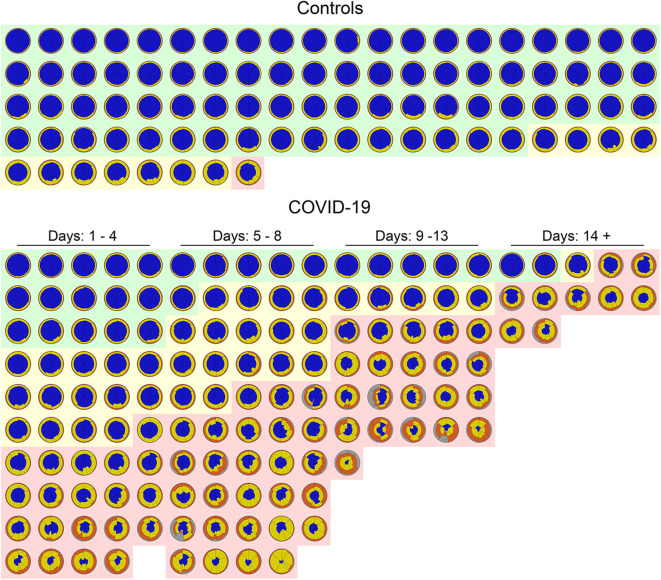
Glyphs of all CT scans of 88 controls (upper panel) and 141 COVID-19 CT scans (lower panel). In the control group, glyphs were sorted by the extent of parenchymal opacities related to ground glass opacities (GGO), CP/LO, and consolidation. Note that just 11 (12%) subjects presented some parenchymal opacities, whereas in just one subject (1%) such opacities presented a Z score higher than 3. Also note that there is a clear predominance of GGO in controls. In COVID-19, glyphs were sorted by days of symptom onset and by the extent of pulmonary involvement with the three classified groups: severe (light red cluster), moderate (light yellow cluster), and mild (light green cluster). Patients' glyphs clearly demonstrate the spectrum of parenchymal abnormalities showing a variety of GGO, CP/LO, and consolidation. Note the predominance of yellow and orange (GGO and CP/LO, respectively) in patients classified as presenting severe pulmonary involvement and with longer time since symptom onset. There are few “normal” subjects (22%) in the COVID-19 database, and therefore only a minority of glyphs are predominantly blue all over the lung.

Figure 7 shows a glyph mosaic of 88 CT scans from the control group and 141 from the 130 COVID-19 patients. The regional distribution of well-aerated regions, GGO, CP/LO, and consolidation can be easily determined in each glyph. The division into the different classes of evolution since the onset of symptoms follows a previous report (4).

## Discussion

We developed a CAD to quantify the extent of PI and to identify the most frequent radiological patterns in COVID-19 pneumonia. For that, a classifier based on a supervised ANN was trained, validated, and tested to classify radiological patterns previously selected and consensually classified by two specialized chest radiologists.

Different ANN architectures were tested using the degree of agreement between the ANN and the radiologists. The best validation performance was determined based on the minimization of the cross-entropy, and the performance of the ANN classifier was evaluated with the confusion matrix of the test set as well as from the ROC curve of each radiological pattern between the ANN and the radiologists' classification (Figure 3).

After establishing the best ANN architecture and characterizing the classifier performance, a CAD was constructed to quantify different radiological opacity patterns in a group of voxels clustered into a regular hexagon grid. Thus, we tested the effect of hexagon dimensions on the performance of the classifier since increasing the dimension would represent more noise due to an expected increase in the dispersion of voxel densities and therefore of the quantiles used as input for the ANN classifier (Figure 4). In fact, such effect was demonstrated, and the hexagon with a radius of 2 mm seemed to be the best dimension for grouping voxels maintaining a good performance of the classifier, although there was a slight reduction in agreement both in overall and within classes (Table 1) when compared to ANN performance in the confusion matrix. However, there is still a very significant and promising agreement and, even with the simplicity of the neural network architecture, the performance of the proposed classifier was quite similar to other more complex classifiers already presented in the literature (5, 6, 12).

The extent of PI has been used to phenotype patients with COVID-19 in profiles with greater involvement, “H” profile, that in general presents with greater elastance and intrapulmonary shunt. These patients also have a higher lung weight and tend to require more aggressive clinical management (13–15).

Our CAD was able to determine the extent of the PI and separate controls from COVID-19 patients with great sensitivity and specificity based on the ROC curve (Figure 5). The most severe cases of COVID-19 patients were more prevalent in the later stages of the disease (symptom onset > 9 days; Figure 7), required care in an ICU and mechanical ventilation, and showed greater case fatality (Table 2). Lung weight was also greater in the most severe COVID-19 pneumonia (Table 2), with GGO, CP/LO, and consolidation representing, altogether, 76 ± 10% of the total lung weight.

The worst performance of our CAD was related to the differentiation between GGO and CP/LO, with the highest misclassification, when the 2-mm-radius hexagon was used, occurring between these two classes. Accordingly, 68 CP/LO cases were misclassified by the CAD as GGO and 76 GGO as CP/LO (Table 1). The overlapping zone between GGO and CP/LO and the important dispersion of voxel densities (Figure 4) are probably related to the presence of GGO in the background of CP/LO (3), contributing to reduced ANN performance between these classes. In addition, it is possible that the separation between GGO and CP/LO depends on the scale used for sampling purposes. In fact, a hexagon with a larger radius, therefore grouping a greater number of voxels, tended to reduce the agreement for GGO (from 81 to 61% for hexagons with 2-mm and 4-mm radii, respectively), whereas it increased the concordance for CP/LO (from 70 to 80% for hexagons of 2-mm and 4-mm radii, respectively).

However, it is important to stress that the classification errors between GGO and CP/LO do not seem to significantly influence the computation of the extent of PI. Thus, it is possible that only more complex methods that use the texture pattern of ROIs, such as convolutional neural networks, can precisely distinguish between GGO and CP/LO (16, 17). Since we opted for a simple and computationally less demanding method, we suggest that the neural network proposed herein may be sufficient to quantitate the extent of COVID-19 pneumonia. In addition, a 70% agreement level is considered to be reasonable in the classification of different radiological standards (5) and was achieved in all radiological patterns examined in our study (18–20).

The mosaic of the glyphs from 88 CT scans from the control group and 141 from the COVID-19 patients summarizes information from 35 gigabytes of CT scan data and clearly demonstrates the difference between these two groups in terms of pulmonary aeration and even severity of PI (Figure 7). Even at this resolution, the glyphs provide a succinct overview of the entire database of subjects and highlight the easiness of pinpointing the intra- and intersubject disease distribution.

Some characteristics of our CAD should be highlighted. The ANN classifier was trained with information extracted from the same database of the image to be processed. This likely mitigated possible bias related to reconstruction algorithms or even artifacts attributed to CT acquisition parameters, such as voltage, amperage, and field of view. In spite of that, the inclusion of more heterogeneous pulmonary opacification patterns in the database should be considered in future studies in order to improve the ANN classifier capacity.

Although the proposed ANN was not tested in the clinical practice scenario, the CT scan images used to train and test the ANN algorithm were quite heterogeneous, using several acquisition protocols and about eight kernels belonging to three different manufacturers. However, further studies evaluating the usefulness of the proposed ANN in radiological practice, as well as studies including non-COVID-19 pneumonia patients, are still necessary for the final assessment of the clinical viability of a CAD platform in the routine of radiology services. In fact, future studies using a similar ANN architecture could be performed to identify highly suspicious COVID-19 chest CT images. Certainly, samples from non-COVID-19 must be included in the database for new ANN training, validation, and test sets.

Furthermore, in the face of the high capacity for information synthesis and easy interpretation of quantitative results, the computational time required for processing a whole-lung CT image is quite low (no more than 60 s). Much of the time expense is still in the lung segmentation stage (2 min in most cases, but reaching more than 10 min in cases where there is consolidation in the subpleural regions). Accordingly, the proposed CAD still needs some improvements in imaging pre-processing to simplify the whole pipeline process and become feasible at the clinical scenario.

Finally, the CAD proposed in the present study seems to be able to identify and quantify the extent of pulmonary involvement, helping to phenotype patients with COVID-19 pneumonia. However, further studies are necessary to investigate the association between the extent of pulmonary involvement and the clinical outcomes or even inflammatory markers.

## Take-Home Message

- The proposed deep learning CAD seems to be able to identify and quantify the extent of pulmonary involvement, helping to phenotype patients with COVID-19 pneumonia.
- Patients with severe COVID-19 pulmonary involvement, as determined by the proposed CAD, presented higher lung weight and C-reactive protein at admission and more frequently required invasive ventilation and intensive care unit hospitalization with higher case fatality.

## Code Availability

CAD will be available upon request to the corresponding author.

## Data Availability Statement

All datasets generated for this study are included in the article.

## Ethics Statement

The studies involving human participants were reviewed and approved by The study was conducted in one-hundred thirty patients with COVID-19, confirmed by RT-PCR, admitted at Hospital Copa Star and Hospital Barra D'Or, Rio de Janeiro, Brazil and at Centro Hospitalar Universitário do Porto (CHUP), Porto, Portugal, and underwent chest CT scans. Eighty-eight healthy subjects who underwent chest CT for clinical purposes and who images were considered nonpathological by radiologists served as controls. Each respective Research Ethics Committee approved the present study (075-DEFI/ 076-CE from CHUP and CAAE – 29496920.8.0000.5262, Institute D'Or), that complied with the current national and international standards. The ethics committee waived the requirement of written informed consent for participation. Written informed consent was not obtained from the individual(s) for the publication of any potentially identifiable images or data included in this article.

## Consent to Participate

As this is a retrospective study without intervention in patients with infectious disease still in hospital or isolation, the consent form was waived by the respective ethics committees.

## Consent for Publication

All authors involved in the present work have read the text and agree to its publication.

## Author Contributions

AC contributed to image processing and analysis of results, statistical evaluation, theoretical development of the neural network, and the computation method of voxel-to-voxel analysis as well as writing of the text and submission of the article. AG contributed to image processing and segmentation, statistical evaluation, and neural network implementation. GW contributed to the determination of image regions of interest, capture and organization of clinical data and draft review. SC and MF contributed to capturing and organization of clinical data and draft review. JP and BG contributed to capturing and organization of clinical data. WS contributed to the determination of image regions of interest and draft review. FB contributed to the results, discussion, and draft review. WZ contributed to draft review. RR contributed to capturing and organization of clinical data, results discussion, and draft review. All authors contributed to the article and approved the submitted version.

## Conflict of Interest

The authors declare that the research was conducted in the absence of any commercial or financial relationships that could be construed as a potential conflict of interest.
